# *tert*-Butyl *N*-benzyl-*N*-[4-(4-fluoro­benzoyl­meth­yl)-2-pyrid­yl]carbamate

**DOI:** 10.1107/S160053680803448X

**Published:** 2008-10-31

**Authors:** Pierre Koch, Dieter Schollmeyer, Stefan Laufer

**Affiliations:** a Institute of Pharmacy, Department of Pharmaceutical and Medicinal Chemistry, Eberhard-Karls-University Tübingen, Auf der Morgenstelle 8, 72076 Tübingen, Germany; b Department of Organic Chemistry, Johannes Gutenberg-University Mainz, Duesbergweg 10-14, D-55099 Mainz, Germany

## Abstract

In the crystal structure of the title compound, C_25_H_25_FN_2_O_3_, the pyridine ring makes dihedral angles of 75.1 (3), 39.4 (3) and 74.6 (3)° with the phenyl ring, the carbamate plane and the 4-fluoro­phenyl ring, respectively. The phenyl ring makes dihedral angles of 77.2 (3) and 23.6 (3)° with the carbamate plane and the 4-fluoro­phenyl ring, respectively. The 4-fluoro­phenyl ring is perpendicular to the carbamate plane, the dihedral angle between them being 89.5 (3)°.

## Related literature

For preparation of the title compound, see: Koch *et al.* (2008*a* ▶). For applications of the vicinal 4-fluoro­phen­yl/pyridin-4-yl pharmacophore in p38 MAP kinase inhibitors, see, for example: Koch *et al.* (2008*a* ▶); for thia­zolopyridines, see: Miwatashi *et al.* (2005 ▶); for pyrazolopyridines, see: Stevens *et al.* (2005 ▶). For a related structure, see: Koch, *et al.* (2008*b* ▶).
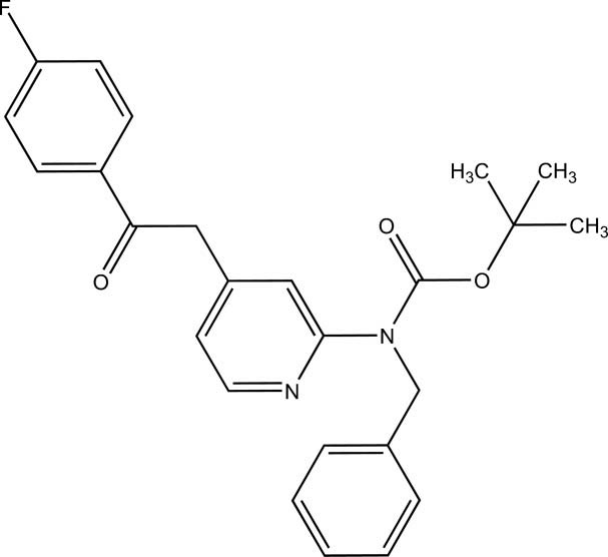


## Experimental

### 

#### Crystal data

C_25_H_25_FN_2_O_3_
*M* *_r_* = 420.47
Monoclinic, 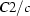

*a* = 38.054 (7) Å
*b* = 7.9320 (6) Å
*c* = 14.589 (3) Å
β = 102.142 (8)°
*V* = 4305.1 (11) Å^3^
*Z* = 8
Cu *K*α radiation
μ = 0.75 mm^−1^
*T* = 193 (2) K
0.35 × 0.30 × 0.18 mm

#### Data collection

Enraf–Nonius CAD-4 diffractometer
Absorption correction: none
4272 measured reflections
4091 independent reflections
2192 reflections with *I* > 2σ(*I*)
*R*_int_ = 0.076
3 standard reflections frequency: 60 min intensity decay: 3%

#### Refinement

*R*[*F* ^2^ > 2σ(*F* ^2^)] = 0.110
*wR*(*F* ^2^) = 0.339
*S* = 1.10
4091 reflections
283 parameters
H-atom parameters constrained
Δρ_max_ = 0.48 e Å^−3^
Δρ_min_ = −0.64 e Å^−3^

### 

Data collection: *CAD-4 Software* (Enraf–Nonius, 1989 ▶); cell refinement: *CAD-4 Software*; data reduction: *CORINC* (Dräger & Gattow, 1971 ▶); program(s) used to solve structure: *SIR97* (Altomare *et al.*, 1999 ▶); program(s) used to refine structure: *SHELXL97* (Sheldrick, 2008 ▶); molecular graphics: *PLATON* (Spek, 2003 ▶); software used to prepare material for publication: *PLATON*.

## Supplementary Material

Crystal structure: contains datablocks I, global. DOI: 10.1107/S160053680803448X/si2118sup1.cif

Structure factors: contains datablocks I. DOI: 10.1107/S160053680803448X/si2118Isup2.hkl

Additional supplementary materials:  crystallographic information; 3D view; checkCIF report

## Figures and Tables

**Fig. 1.**
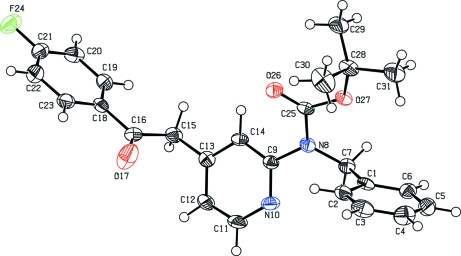
View of compound I. Displacement ellipsoids are drawn at the 50% probability level. H atoms are depicted as circles of arbitrary size.

## supplementary crystallographic information

### Comment

The title compound, (I), was obtained as an intermediate in the synthesis of 2-alkylsulfanyl-5-(2-aminopyridin-4-yl)-4-(4-fluorophenyl)imidazoles as potent p38 MAP kinase inhibitors (Koch *et al.* 2008*a*).

The vicinal 4-fluorophenyl/pyridin-4-yl system is a pharmacophore in different p38 MAP kinase inhibitors, like the imidazolopyridines (Koch *et al.* 2008*a*,*b*) and related pyridine compounds (Miwatashi *et al.* 2005), (Stevens *et al.* 2005).

In the crystal structure of the title compound I, Fig. 1, the pyridine ring makes dihedral angles of 75.1 (3)°, 39.4 (3)° and 74.6 (3)° to the phenyl ring (C1–C6), the carbamate plane and the 4-fluorophenyl ring (C18–C23), respectively. The phenyl ring (C1–C6) makes dihedral angles of 77.2 (3)° and 23.6 (3)° to the carbamate plane and the 4-fluorophenyl ring (C18–C23), respectively. The 4-fluorophenyl ring (C18–C23) is perpendicular to the carbamate acid plane, the dihedral angle between them is 89.5 (3)°.

The 4-fluorophenyl group is rotating away from the pyridine ring system compared to the recently published crystal structure of methyl 4-(5-(4-fluorophenyl)-4-(pyridin-4-yl)-1*H*-imidazol-2-ylthio)butanoate (Koch *et al.* 2008*b*).

### Experimental

*tert*-Butyl *N*-benzyl-*N*-(4-methylpyridin-2-yl)carbamate (6.27 g, 21.0 mmol) and ethyl 4-fluorobenzoate (3.89 g, 23.1 mmol) were dissolved in dry THF (60 ml) under argon atmosphere. The solution was cooled to 273 K and NaHMDS (21.0 ml, 42.0 mmol, 2 *M* in THF) was added dropwise. The mixture was allowed to stir at this temperature for 1 h and additional 2.5 h at room temperature. The reaction was quenched with saturated NH_4_Cl solution, EtOAc was added and the mixture was extracted twice with water. The organic layer was dried (sodium sulfate) and concentrated *in vacuo*. The crude product was purified by flash chromatography (silica gel, petroleum ether/ethylacetate 5–1 to 3–1) to yield 5.40 g (61%) of I as colourless crystals (Koch *et al.* 2008*a*).

### Refinement

Hydrogen atoms attached to carbons were placed at calculated positions with C—H = 0.95 Å (aromatic) or 0.98–0.99 Å (*sp*^3^ C-atom). All H atoms were refined in the riding-model approximation with isotropic displacement parameters (set at 1.2–1.5 times of the *U*_eq_ of the parent atom).

### Crystal data

**Table:**

C_25_H_25_FN_2_O_3_	*F*(000) = 1776
*M**_r_* = 420.47	*D*_x_ = 1.297 Mg m^−^^3^
Monoclinic, *C*2/*c*	Cu *K*α radiation, λ = 1.54178 Å
Hall symbol: -C 2yc	Cell parameters from 25 reflections
*a* = 38.054 (7) Å	θ = 15–28°
*b* = 7.9320 (6) Å	µ = 0.75 mm^−^^1^
*c* = 14.589 (3) Å	*T* = 193 K
β = 102.142 (8)°	Plate, colourless
*V* = 4305.1 (11) Å^3^	0.35 × 0.30 × 0.18 mm
*Z* = 8	

### Data collection

**Table:**

Enraf–Nonius CAD-4 diffractometer	*R*_int_ = 0.076
Radiation source: rotating anode	θ_max_ = 70.1°, θ_min_ = 2.4°
graphite	*h* = −46→45
ω/2θ scans	*k* = −9→0
4272 measured reflections	*l* = 0→17
4091 independent reflections	3 standard reflections every 60 min
2192 reflections with *I* > 2σ(*I*)	intensity decay: 3%

### Refinement

**Table:**

Refinement on *F*^2^	Primary atom site location: structure-invariant direct methods
Least-squares matrix: full	Secondary atom site location: difference Fourier map
*R*[*F*^2^ > 2σ(*F*^2^)] = 0.110	Hydrogen site location: inferred from neighbouring sites
*wR*(*F*^2^) = 0.339	H-atom parameters constrained
*S* = 1.10	*w* = 1/[σ^2^(*F*_o_^2^) + (0.182*P*)^2^] where *P* = (*F*_o_^2^ + 2*F*_c_^2^)/3
4091 reflections	(Δ/σ)_max_ < 0.001
283 parameters	Δρ_max_ = 0.48 e Å^−^^3^
0 restraints	Δρ_min_ = −0.64 e Å^−^^3^

### Special details

**Table:**

Experimental. No absorption correction was applied because of irregular crystal shape and low crystal quality. The crystal diffracted only very weak (less the 55% observed reflections).
Geometry. All e.s.d.'s (except the e.s.d. in the dihedral angle between two l.s. planes) are estimated using the full covariance matrix. The cell e.s.d.'s are taken into account individually in the estimation of e.s.d.'s in distances, angles and torsion angles; correlations between e.s.d.'s in cell parameters are only used when they are defined by crystal symmetry. An approximate (isotropic) treatment of cell e.s.d.'s is used for estimating e.s.d.'s involving l.s. planes.
Refinement. Refinement of *F*^2^ against ALL reflections. The weighted *R*-factor *wR* and goodness of fit *S* are based on *F*^2^, conventional *R*-factors *R* are based on *F*, with *F* set to zero for negative *F*^2^. The threshold expression of *F*^2^ > σ(*F*^2^) is used only for calculating *R*-factors(gt) *etc*. and is not relevant to the choice of reflections for refinement. *R*-factors based on *F*^2^ are statistically about twice as large as those based on *F*, and *R*- factors based on ALL data will be even larger.

### Fractional atomic coordinates and isotropic or equivalent isotropic displacement parameters (Å^2^)

**Table:**

	*x*	*y*	*z*	*U*_iso_*/*U*_eq_	
C1	0.30976 (15)	0.4803 (7)	0.3672 (4)	0.0242 (12)	
C2	0.33725 (17)	0.5800 (8)	0.4143 (4)	0.0324 (14)	
H2	0.3612	0.5383	0.4269	0.039*	
C3	0.33038 (19)	0.7421 (8)	0.4438 (5)	0.0406 (16)	
H3	0.3495	0.8094	0.4773	0.049*	
C4	0.2957 (2)	0.8035 (9)	0.4240 (5)	0.0422 (17)	
H4	0.2909	0.9137	0.4437	0.051*	
C5	0.26812 (18)	0.7057 (9)	0.3759 (4)	0.0386 (16)	
H5	0.2443	0.7486	0.3619	0.046*	
C6	0.27507 (16)	0.5439 (8)	0.3476 (4)	0.0297 (13)	
H6	0.2559	0.4764	0.3146	0.036*	
C7	0.31548 (15)	0.3018 (8)	0.3346 (4)	0.0265 (13)	
H7A	0.2945	0.2325	0.3406	0.032*	
H7B	0.3163	0.3058	0.2672	0.032*	
N8	0.34806 (12)	0.2177 (6)	0.3852 (3)	0.0273 (11)	
C9	0.37651 (15)	0.1822 (7)	0.3379 (4)	0.0241 (12)	
N10	0.38074 (14)	0.3047 (6)	0.2779 (3)	0.0326 (12)	
C11	0.40604 (19)	0.2738 (9)	0.2270 (5)	0.0440 (18)	
H11	0.4097	0.3582	0.1838	0.053*	
C12	0.42649 (17)	0.1328 (9)	0.2326 (4)	0.0389 (16)	
H12	0.4433	0.1178	0.1934	0.047*	
C13	0.42200 (15)	0.0098 (8)	0.2982 (4)	0.0268 (13)	
C14	0.39684 (15)	0.0377 (7)	0.3519 (4)	0.0240 (12)	
H14	0.3936	−0.0418	0.3981	0.029*	
C15	0.44316 (16)	−0.1512 (8)	0.3080 (4)	0.0297 (13)	
H15A	0.4419	−0.1996	0.2448	0.036*	
H15B	0.4319	−0.2328	0.3443	0.036*	
C16	0.48232 (17)	−0.1287 (8)	0.3558 (5)	0.0341 (15)	
O17	0.49559 (13)	0.0077 (6)	0.3700 (5)	0.0666 (18)	
C18	0.50420 (15)	−0.2837 (8)	0.3851 (4)	0.0268 (13)	
C19	0.49111 (16)	−0.4454 (8)	0.3620 (4)	0.0279 (13)	
H19	0.4676	−0.4597	0.3249	0.034*	
C20	0.51188 (18)	−0.5852 (8)	0.3923 (4)	0.0337 (15)	
H20	0.5030	−0.6957	0.3766	0.040*	
C21	0.54570 (18)	−0.5608 (8)	0.4458 (4)	0.0332 (14)	
C22	0.55982 (19)	−0.4035 (9)	0.4691 (5)	0.0413 (17)	
H22	0.5835	−0.3907	0.5054	0.050*	
C23	0.53868 (16)	−0.2635 (8)	0.4385 (4)	0.0325 (14)	
H23	0.5479	−0.1535	0.4541	0.039*	
F24	0.56589 (11)	−0.6962 (5)	0.4773 (3)	0.0487 (11)	
C25	0.35189 (15)	0.1753 (7)	0.4796 (3)	0.0213 (12)	
O26	0.37827 (11)	0.1119 (5)	0.5264 (3)	0.0299 (10)	
O27	0.32169 (10)	0.2188 (5)	0.5079 (2)	0.0260 (9)	
C28	0.32262 (16)	0.2339 (8)	0.6097 (3)	0.0307 (14)	
C29	0.32429 (19)	0.0591 (10)	0.6534 (4)	0.0440 (18)	
H29A	0.3033	−0.0063	0.6227	0.066*	
H29B	0.3245	0.0693	0.7204	0.066*	
H29C	0.3462	0.0018	0.6451	0.066*	
C30	0.3536 (2)	0.3456 (11)	0.6559 (5)	0.053 (2)	
H30A	0.3763	0.2953	0.6482	0.080*	
H30B	0.3537	0.3566	0.7228	0.080*	
H30C	0.3509	0.4573	0.6265	0.080*	
C31	0.28696 (18)	0.3212 (10)	0.6091 (4)	0.0443 (18)	
H31A	0.2861	0.4284	0.5752	0.066*	
H31B	0.2847	0.3428	0.6737	0.066*	
H31C	0.2672	0.2488	0.5779	0.066*	

### Atomic displacement parameters (Å^2^)

**Table:**

	*U*^11^	*U*^22^	*U*^33^	*U*^12^	*U*^13^	*U*^23^
C1	0.032 (3)	0.026 (3)	0.018 (3)	−0.006 (2)	0.012 (2)	0.000 (2)
C2	0.034 (3)	0.027 (3)	0.039 (3)	−0.003 (3)	0.014 (3)	0.006 (3)
C3	0.052 (4)	0.029 (4)	0.042 (4)	−0.015 (3)	0.013 (3)	−0.005 (3)
C4	0.061 (5)	0.028 (4)	0.042 (4)	0.010 (3)	0.021 (3)	−0.005 (3)
C5	0.039 (4)	0.045 (4)	0.034 (3)	0.010 (3)	0.014 (3)	0.005 (3)
C6	0.035 (3)	0.033 (3)	0.023 (3)	0.004 (3)	0.009 (2)	0.002 (3)
C7	0.031 (3)	0.036 (3)	0.013 (2)	0.002 (3)	0.006 (2)	−0.001 (2)
N8	0.029 (3)	0.037 (3)	0.018 (2)	0.003 (2)	0.0098 (19)	0.002 (2)
C9	0.027 (3)	0.027 (3)	0.019 (2)	−0.002 (2)	0.006 (2)	0.002 (2)
N10	0.044 (3)	0.029 (3)	0.027 (3)	0.007 (2)	0.015 (2)	0.009 (2)
C11	0.054 (4)	0.046 (4)	0.040 (4)	0.013 (3)	0.031 (3)	0.021 (3)
C12	0.042 (4)	0.049 (4)	0.035 (3)	0.012 (3)	0.028 (3)	0.011 (3)
C13	0.029 (3)	0.029 (3)	0.022 (3)	−0.001 (3)	0.005 (2)	−0.003 (2)
C14	0.032 (3)	0.025 (3)	0.015 (2)	−0.003 (2)	0.006 (2)	0.001 (2)
C15	0.037 (3)	0.030 (3)	0.026 (3)	0.003 (3)	0.015 (2)	0.000 (3)
C16	0.036 (3)	0.028 (3)	0.040 (3)	0.000 (3)	0.011 (3)	0.012 (3)
O17	0.042 (3)	0.028 (3)	0.118 (5)	−0.006 (2)	−0.011 (3)	0.009 (3)
C18	0.027 (3)	0.030 (3)	0.027 (3)	−0.001 (2)	0.013 (2)	0.003 (3)
C19	0.031 (3)	0.032 (3)	0.023 (3)	0.000 (3)	0.011 (2)	−0.004 (3)
C20	0.051 (4)	0.022 (3)	0.033 (3)	0.002 (3)	0.020 (3)	−0.001 (3)
C21	0.042 (4)	0.035 (4)	0.026 (3)	0.014 (3)	0.016 (3)	0.004 (3)
C22	0.035 (4)	0.050 (5)	0.039 (4)	0.006 (3)	0.008 (3)	0.009 (3)
C23	0.037 (3)	0.030 (3)	0.033 (3)	−0.001 (3)	0.013 (3)	0.005 (3)
F24	0.062 (3)	0.040 (2)	0.045 (2)	0.022 (2)	0.015 (2)	0.0098 (19)
C25	0.031 (3)	0.021 (3)	0.014 (2)	−0.007 (2)	0.008 (2)	−0.004 (2)
O26	0.032 (2)	0.040 (3)	0.0165 (19)	0.0031 (19)	0.0030 (16)	0.0027 (18)
O27	0.033 (2)	0.032 (2)	0.0157 (18)	−0.0009 (18)	0.0114 (16)	−0.0026 (17)
C28	0.042 (3)	0.043 (4)	0.009 (2)	−0.005 (3)	0.011 (2)	−0.007 (2)
C29	0.042 (4)	0.070 (5)	0.022 (3)	−0.001 (4)	0.010 (3)	0.013 (3)
C30	0.055 (5)	0.071 (6)	0.035 (4)	−0.017 (4)	0.012 (3)	−0.023 (4)
C31	0.053 (4)	0.059 (5)	0.025 (3)	0.002 (4)	0.019 (3)	−0.003 (3)

### Geometric parameters (Å, °)

**Table:**

C1—C2	1.374 (8)	O27—C28	1.484 (6)
C1—C6	1.385 (8)	C28—C30	1.515 (9)
C1—C7	1.525 (8)	C28—C29	1.521 (9)
C2—C3	1.398 (9)	C28—C31	1.522 (9)
C3—C4	1.378 (10)	C2—H2	0.9500
C4—C5	1.375 (10)	C3—H3	0.9500
C5—C6	1.391 (9)	C4—H4	0.9500
C7—N8	1.464 (7)	C5—H5	0.9500
N8—C25	1.396 (6)	C6—H6	0.9500
N8—C9	1.429 (7)	C7—H7A	0.9900
C9—N10	1.340 (7)	C7—H7B	0.9900
C9—C14	1.374 (8)	C11—H11	0.9500
N10—C11	1.356 (7)	C12—H12	0.9500
C11—C12	1.355 (9)	C14—H14	0.9500
C12—C13	1.403 (8)	C15—H15A	0.9900
C13—C14	1.376 (7)	C15—H15B	0.9900
C13—C15	1.500 (8)	C19—H19	0.9500
C15—C16	1.517 (9)	C20—H20	0.9500
C16—O17	1.194 (8)	C22—H22	0.9500
C16—C18	1.496 (8)	C23—H23	0.9500
C18—C23	1.387 (8)	C29—H29A	0.9800
C18—C19	1.391 (8)	C29—H29B	0.9800
C19—C20	1.380 (8)	C29—H29C	0.9800
C20—C21	1.371 (9)	C30—H30A	0.9800
C21—F24	1.344 (7)	C30—H30B	0.9800
C21—C22	1.373 (10)	C30—H30C	0.9800
C22—C23	1.389 (9)	C31—H31A	0.9800
C25—O26	1.199 (7)	C31—H31B	0.9800
C25—O27	1.345 (6)	C31—H31C	0.9800
			
C2—C1—C6	119.1 (6)	C4—C3—H3	120.00
C2—C1—C7	123.2 (5)	C3—C4—H4	120.00
C6—C1—C7	117.8 (5)	C5—C4—H4	120.00
C1—C2—C3	120.7 (6)	C4—C5—H5	120.00
C4—C3—C2	119.6 (6)	C6—C5—H5	120.00
C5—C4—C3	120.2 (6)	C1—C6—H6	120.00
C4—C5—C6	120.0 (6)	C5—C6—H6	120.00
C1—C6—C5	120.5 (6)	N8—C7—H7A	108.00
N8—C7—C1	115.3 (5)	N8—C7—H7B	108.00
C25—N8—C9	119.8 (5)	C1—C7—H7A	109.00
C25—N8—C7	120.7 (4)	C1—C7—H7B	108.00
C9—N8—C7	119.5 (4)	H7A—C7—H7B	107.00
N10—C9—C14	124.1 (5)	N10—C11—H11	117.00
N10—C9—N8	112.3 (5)	C12—C11—H11	117.00
C14—C9—N8	123.5 (5)	C11—C12—H12	121.00
C9—N10—C11	115.0 (5)	C13—C12—H12	121.00
C12—C11—N10	125.5 (6)	C9—C14—H14	120.00
C11—C12—C13	117.7 (5)	C13—C14—H14	120.00
C14—C13—C12	118.4 (5)	C13—C15—H15A	109.00
C14—C13—C15	120.5 (5)	C13—C15—H15B	109.00
C12—C13—C15	121.1 (5)	C16—C15—H15A	109.00
C9—C14—C13	119.1 (5)	C16—C15—H15B	109.00
C13—C15—C16	113.6 (5)	H15A—C15—H15B	108.00
O17—C16—C18	120.4 (6)	C18—C19—H19	120.00
O17—C16—C15	121.7 (6)	C20—C19—H19	120.00
C18—C16—C15	118.0 (5)	C19—C20—H20	121.00
C23—C18—C19	119.4 (6)	C21—C20—H20	121.00
C23—C18—C16	118.0 (6)	C21—C22—H22	121.00
C19—C18—C16	122.6 (5)	C23—C22—H22	121.00
C20—C19—C18	120.8 (6)	C18—C23—H23	120.00
C21—C20—C19	118.4 (6)	C22—C23—H23	120.00
F24—C21—C20	118.9 (6)	C28—C29—H29A	109.00
F24—C21—C22	118.4 (6)	C28—C29—H29B	109.00
C20—C21—C22	122.7 (6)	C28—C29—H29C	109.00
C21—C22—C23	118.5 (6)	H29A—C29—H29B	110.00
C18—C23—C22	120.3 (6)	H29A—C29—H29C	109.00
O26—C25—O27	126.9 (5)	H29B—C29—H29C	109.00
O26—C25—N8	124.3 (5)	C28—C30—H30A	109.00
O27—C25—N8	108.8 (5)	C28—C30—H30B	110.00
C25—O27—C28	119.0 (4)	C28—C30—H30C	109.00
O27—C28—C30	110.2 (5)	H30A—C30—H30B	109.00
O27—C28—C29	109.6 (5)	H30A—C30—H30C	109.00
C30—C28—C29	112.8 (6)	H30B—C30—H30C	109.00
O27—C28—C31	101.4 (5)	C28—C31—H31A	109.00
C30—C28—C31	110.2 (6)	C28—C31—H31B	109.00
C29—C28—C31	112.1 (5)	C28—C31—H31C	109.00
C1—C2—H2	120.00	H31A—C31—H31B	109.00
C3—C2—H2	120.00	H31A—C31—H31C	109.00
C2—C3—H3	120.00	H31B—C31—H31C	110.00
			
C6—C1—C2—C3	1.3 (8)	C12—C13—C15—C16	73.5 (7)
C7—C1—C2—C3	−179.1 (5)	C13—C15—C16—O17	−11.2 (9)
C1—C2—C3—C4	−1.1 (9)	C13—C15—C16—C18	168.9 (5)
C2—C3—C4—C5	0.2 (10)	O17—C16—C18—C23	7.5 (9)
C3—C4—C5—C6	0.5 (10)	C15—C16—C18—C23	−172.6 (5)
C2—C1—C6—C5	−0.6 (8)	O17—C16—C18—C19	−173.4 (7)
C7—C1—C6—C5	179.8 (5)	C15—C16—C18—C19	6.5 (8)
C4—C5—C6—C1	−0.3 (9)	C23—C18—C19—C20	0.6 (8)
C2—C1—C7—N8	22.7 (7)	C16—C18—C19—C20	−178.4 (5)
C6—C1—C7—N8	−157.7 (5)	C18—C19—C20—C21	0.0 (8)
C1—C7—N8—C25	66.5 (7)	C19—C20—C21—F24	178.7 (5)
C1—C7—N8—C9	−112.1 (5)	C19—C20—C21—C22	−0.9 (9)
C25—N8—C9—N10	−141.6 (5)	F24—C21—C22—C23	−178.5 (5)
C7—N8—C9—N10	37.1 (7)	C20—C21—C22—C23	1.1 (10)
C25—N8—C9—C14	39.1 (8)	C19—C18—C23—C22	−0.5 (9)
C7—N8—C9—C14	−142.3 (6)	C16—C18—C23—C22	178.6 (6)
C14—C9—N10—C11	2.8 (9)	C21—C22—C23—C18	−0.4 (9)
N8—C9—N10—C11	−176.5 (6)	C9—N8—C25—O26	1.4 (9)
C9—N10—C11—C12	0.0 (11)	C7—N8—C25—O26	−177.2 (6)
N10—C11—C12—C13	−1.9 (12)	C9—N8—C25—O27	−179.2 (5)
C11—C12—C13—C14	1.0 (10)	C7—N8—C25—O27	2.2 (7)
C11—C12—C13—C15	179.1 (6)	O26—C25—O27—C28	17.1 (8)
N10—C9—C14—C13	−3.7 (9)	N8—C25—O27—C28	−162.3 (5)
N8—C9—C14—C13	175.5 (5)	C25—O27—C28—C30	51.0 (7)
C12—C13—C14—C9	1.6 (8)	C25—O27—C28—C29	−73.6 (6)
C15—C13—C14—C9	−176.5 (5)	C25—O27—C28—C31	167.7 (5)
C14—C13—C15—C16	−108.4 (6)		
